# Fasciclin-like arabinogalactan proteins, PtFLAs, play important roles in GA-mediated tension wood formation in *Populus*

**DOI:** 10.1038/s41598-017-06473-9

**Published:** 2017-07-21

**Authors:** Haihai Wang, Yanli Jin, Cuiting Wang, Bei Li, Chunmei Jiang, Zhencang Sun, Zhiping Zhang, Fanjing Kong, Hongxia Zhang

**Affiliations:** 1 National Key Laboratory of Plant Molecular Genetics, Shanghai Institute of Plant Physiology and Ecology, Chinese Academy of Sciences, 300 Fenglin Road, Shanghai, 200032 China; 2 College of Agriculture, Ludong University, 186 Hongqizhong Road, Yantai, 264025 China; 3 Institute of Mineral Resources, CAGS, MLR Key Laboratory of Saline Lake Resources and Environments, Beijing, 100037 China; 4 Shanghai Key Laboratory of Bio-Energy Crops, School of Life Sciences, Shanghai University, Shanghai, 200444 China

## Abstract

In *Populus*, the transcripts of fasciclin-like arabinogalactan proteins (FLAs) are accumulated in tension wood (TW) xylem, however their biological functions in TW formation are largely unknown. In this work, we demonstrated that PtFLA6, one of poplar TW-associated PtFLAs, was abundantly expressed in TW, and mainly localized in differentiating G-fibers. The bended stems of *PtFLA6* antisense transgenic poplar showed decreased transcripts of *PtFLAs*, including *PtFLA6*, and reduced PtFLA6 like proteins, leading to inhibited TW differentiation and formation. We also showed that gibberellin A3 (GA_3_) was enriched in the xylem of TW side, accompanied with a lowered level of PtRGA1, a poplar DELLA protein. When GA_3_ biosynthesis was restrained in the bended poplar stems by a GA biosynthesis inhibitor (daminozide), TW formation was obviously repressed, as a result of restricted PtRGA1 degradation, and reduced PtFLA6 like proteins and *PtFLA* expression. Further studies indicated that *PtFLAs* were negatively regulated by PtRGA1. This study suggests that PtFLAs play important roles in the poplar TW formation, possibly regulated by GA signaling.

## Introduction

As perennial plants, trees are often inclined or bended when they encounter gravitation or mechanical stresses such as wind, snow, and growth on slope. In response to the perception of gravity or mechanical stimuli, woody plants form an abnormal tissue, named reaction wood (RW). RW, formed on the upper side of leaning or bended stems in angiosperms, is called tension wood (TW)^1^, which generates a tensile force in order to pull the displaced stem to its original position^2^.

TW is characterized by a higher proportion of fibers and a lower proportion of vessel elements^3^. The most striking modification is the presence of an additional thick gelatinous cell wall layer (G-layer) in the TW fibers. The G-layer is a layer of the secondary wall (replacing most of the S2 and the entire S3 layer) and is mainly composed of crystalline cellulose^1, 4^. Although TW is considered to reduce the wood quality and be a nuisance in wood processing and paper industry, it is beneficial to increase the glucose yields in biofuel industry^5^. Therefore, study on the molecular mechanism of TW formation is becoming more scientifically and commercially meaningful.

Tension wood formation has been thought to be regulated by plant hormones, such as auxin, ethylene and gibberellins (GAs). During TW formation, expressions of several *Aux*/*IAA* genes were changed^6^ and the auxin transporter PIN3 transported auxin toward the cambium in the TW side to trigger TW formation^7^. However the balance of endogenous auxin level was not significantly altered^8^, which indicated that auxin may not directly regulate TW formation. Ethylene has been confirmed as a key regulator in poplar TW formation. In the gravity-induced tension wood tissues of *Populus*, both the expression of *PttACO1* which encodes a 1-aminocyclopropane-1-carboxylic acid (ACC) oxidase in ethylene biosynthesis pathway, and the enzyme activity of ACC were increased^9^. On the other hand, in ethylene insensitive transgenic trees and those treated with ethylene perception inhibitor [1-methylcyclopropene (1-MCP)], TW formation was obviously repressed^10^. GA is another kind of plant hormones regulating tension wood formation. Application of GA could induce TW formation in angiosperm trees^11^. A recent report suggested that GA controlled auxin transport during TW formation, and that GA stimulated TW formation was sensitive to ARK2 levels^7^. However the exact regulatory mechanism of GA-mediated TW formation is still unclear.

FLAs (fasciclin-like AGPs), containing one or more fasciclin domain(s)^12^, belong to the arabinogalactan proteins (AGPs), and are subdivided into four groups named A to D^13^. FLAs played important roles in seed coat mucilage, normal cell expansion, stem biomechanics and cell wall architecture in *Arabidopsis*, and fiber initiation and elongation in cotton^14–17^. Interestingly, a group of FLAs belonging to subgroup A were highly up-regulated in the TW tissues compared with that in the opposite wood (OW) tissues of poplar^7, 18–20^. In addition, a willow FLA gene *SxFLA12* was also specifically expressed in developing TW^7^. All these reports indicate that FLAs may play important roles during TW formation of woody plants. However, the biological functions of FLAs in TW formation are not well illustrated yet.

In a previous study, we found that PtFLAs affected the poplar stem biomechanics by altering cell wall compositions in *Populus* ^21^. In the present work, we further investigated the biological function of PtFLAs in mechanical stress induced TW formation using the bended *PtFLA6* antisense transgenic poplar plants. Our results suggest that PtFLAs play important roles in the TW formation of poplar.

## Results

### PtFLA6 is specifically expressed in TW

**Figure 1:**
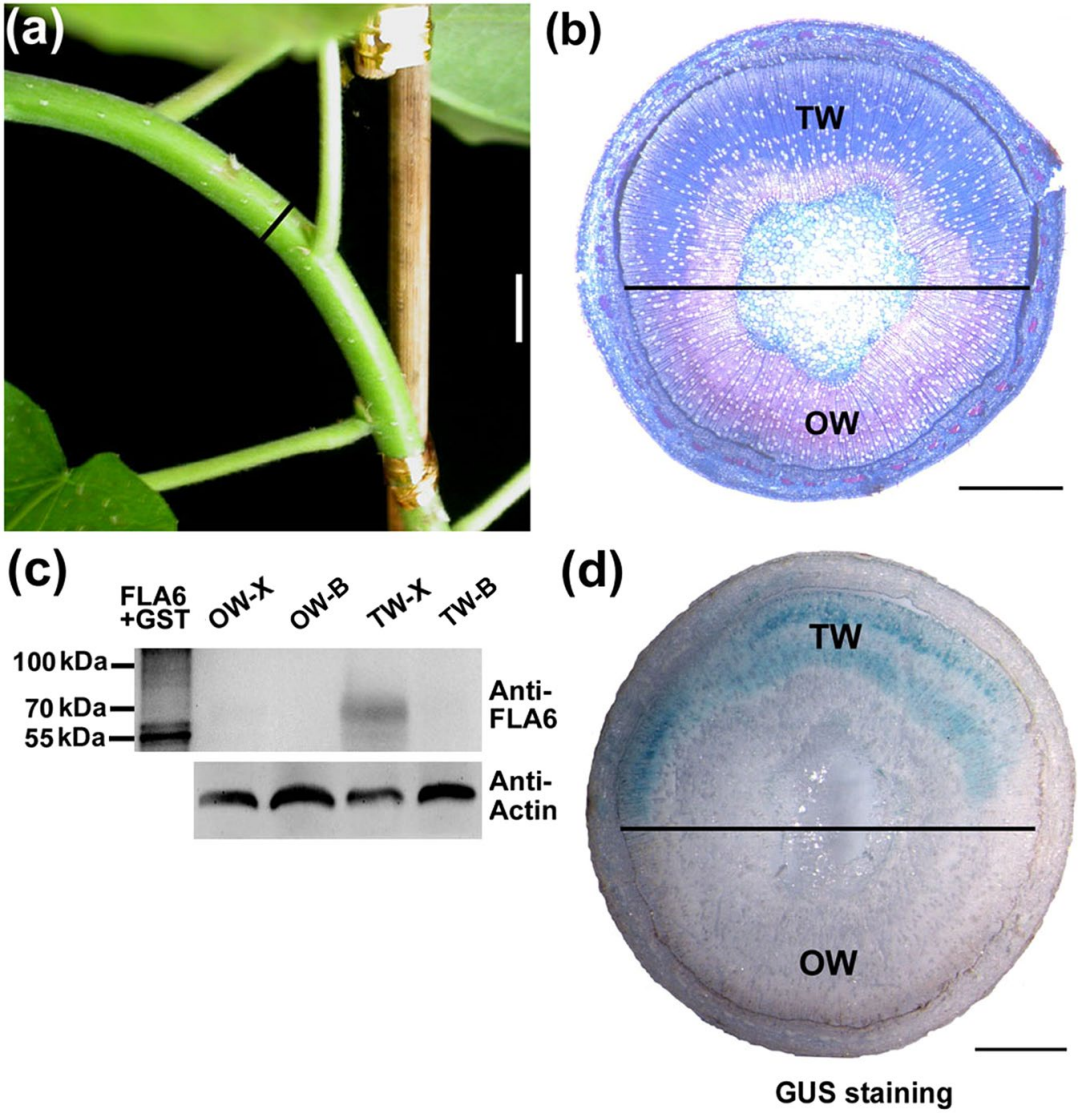
PtFLA6 expression analyses during TW formation. (**a**) TW induction by mechanical stress. Two-month-old wild type poplar plants (Shanxin yang) grown in greenhouse were bended for two weeks. The black vertical bar indicates the part to be taken for analyses. Scale bar = 1 cm. (**b**) Sections of bended stems were stained with safranin-O and astra-blue. TW was stained into blue. The horizontal line indicates the division between TW side and OW side. TW, tension wood; OW, opposite wood. Scale bar = 1 mm. (**c**) Western blotting analysis of PtFLA6 protein during TW formation. A total amount of 15 µg proteins was separated by SDS-PAGE and hybridized with anti-PtFLA6 antibodies (Anti-FLA6) or plant actin antibodies (Anti-Actin). FLA6 + GST, fusion protein for antibody production; OW-X, xylem tissues in the OW side; OW-B, the bark tissues of OW side; TW-X, xylem tissues in the TW side; TW-B, the bark tissues of TW side. (**d**) *PtFLA6* promoter activity analysis during TW formation. Sections from the bended site of *PtFLA6* promoter-GUS transgenic plants were used for GUS staining. Scale bar = 1 mm.

TW formation can be induced by gravitational stimulus^3, 4^, as well as by mechanical stress^22^. In this study, we bended the stems of poplar plants grown in greenhouse to induce TW formation (Fig. 1a). As expected, obvious TW was produced in the upper side of the bended stems and stained into blue color by safranin/astra blue double staining; and OW in the lower side remained in red color (Fig. 1b).

It has been reported that a group of FLA genes in poplar (*PopFLA1–10*, also named *PtFLA12o*, -*K*, -*i*, -*Q*, -*S*, -*D*, -*V*, -*u*, and -*G*) were highly up-regulated in tension wood^18, 19^, implying their important but unclear functions in TW formation. Based on the nucleotide sequences of *PopFLA1–10*, we extracted ten *FLAs* from the Joint Genome Initiative poplar database (http://genome.jgi.doe.gov/Poptr1_1/Poptr1_1.home.html) and named them as *PtFLA1–10*, respectively. All *PtFLAs* share very high similarity, and *PtFLA2* and *PtFLA3* have exactly the same nucleotide sequence^21^. We then confirmed the expression pattern of *PtFLAs* during TW formation by qRT-PCR analysis (Figure S1). The results showed that all *PtFLAs* were specifically expressed in the xylem tissues of vertically grown stems under normal growth condition in the greenhouse. When the stems were bended to induce TW fromation, *PtFLAs* were also mainly expressed in the xylem tissues in both TW and OW sides. However, all *PtFLAs* were highly expressed in the xylem tissues of the TW side compared to that of the OW side or the xylem tissues of normally grown stems. These results indicate that *PtFLAs* are up-regulated during TW formation to play some particular roles.

To understand the roles of *PtFLAs* in mechanical stress induced TW formation, we selected PtFLA6 with the most specific and abundant expression in TW for further study^18, 19^. We firstly confirmed the expression pattern of PtFLA6 during TW formation by Western blotting analysis. PtFLA6 proteins were predominantly accumulated in the xylem tissues of the TW side compared to that of the OW side (Fig. 1c), in consistence with its gene expression pattern (Figure S1). To further detect the detailed expression of *PtFLA6* during TW formation, we then carried out promoter-GUS analysis. The promoter region (~1.5 kb) of *PtFLA6* was cloned and used to construct the *ProPtFLA6*::*GUS* vector for poplar transformation. Two-month-old transgenic plants grown in greenhouse were bended for two weeks to induce TW formation. GUS staining showed that *PtFLA6* promoter was mainly activated in the TW tissues compared with the phloem and OW tissues (Fig. 1d). These results reveal that PtFLA6 is a classical TW-associated protein.

### PtFLA6 proteins are predominantly accumulated in differentiating G-fiber cells

**Figure 2:**
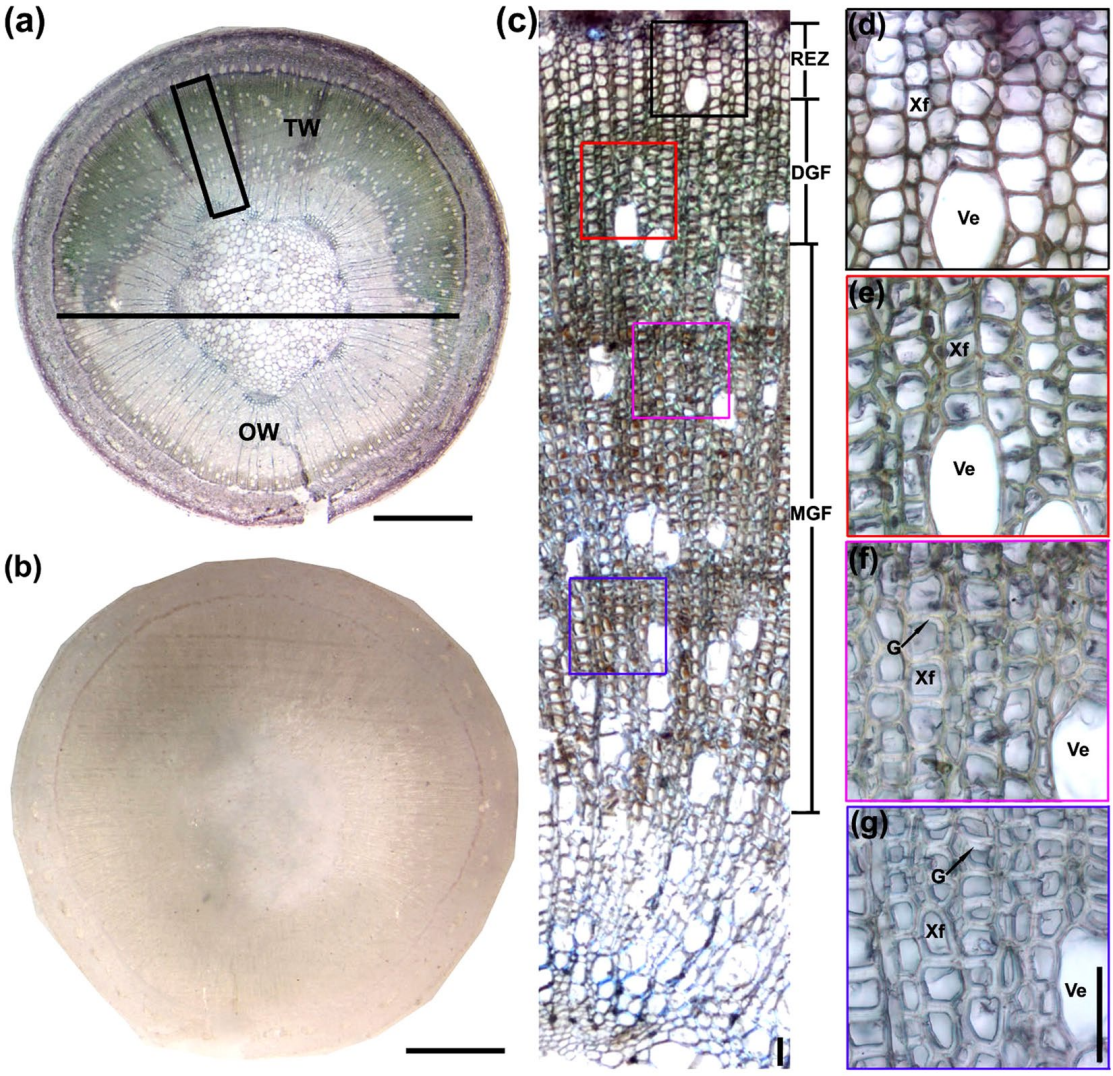
Immunohistochemical analysis of PtFLA6 during TW formation. (**a**,**b**) Cross-sections of the bended stems of the wild type poplar plants were hybridized with anti-PtFLA6 antibodies (**a**) or pre-immune IgG as control (**b**). Green-brown signals of PtFLA6 were specifically detected in the TW. The horizontal line indicates the division between TW side and OW side. TW, tension wood; OW, opposite wood. Scale bar = 1 mm. (**c**) A higher magnification of the framed area in (**a**). REZ, radial expansion zone; DGF, differentiating G-fiber; MGF, mature G-fiber. (**d**–**g**) Higher magnification images of the framed areas in (**c**). PtFLA6 accumulation was decreased as the G-fiber maturing. Ve, vessel cell; Xf, xylem fiber cell. Scale bars = 25 μm.

Proteins only play functions in particular tissues and cells. To observe the precise tissue and cellular locations of PtFLA6 proteins during TW formation, immunohistochemical localization using PtFLA6 antibodies was carried out. Compared with the phloem and OW tissues, high density of green-brown signals representing PtFLA6 proteins were detected in the TW tissues (Fig. 2a), whereas no staining was observed in the control samples (Fig. 2b). Further analyses revealed that PtFLA6 proteins were abundantly accumulated in the differentiating G-fibers (DGFs) undergoing G-layer formation, and in the mature G-fibers (MGFs) with obvious G-layer (Fig. 2c). This observation was more distinct under higher magnification of the images. PtFLA6 signaling was weaker in the radial expansion zone (REZ) (Fig. 2d), but became stronger in the cell wall of DGF (Fig. 2e), and gradually reduced as the G-fibers matured (Fig. 2f,g). In addition, PtFLA6 proteins were mainly present in the outer wall of MGF other than in the G-layer (Fig. 2f,g).

**Figure 3:**
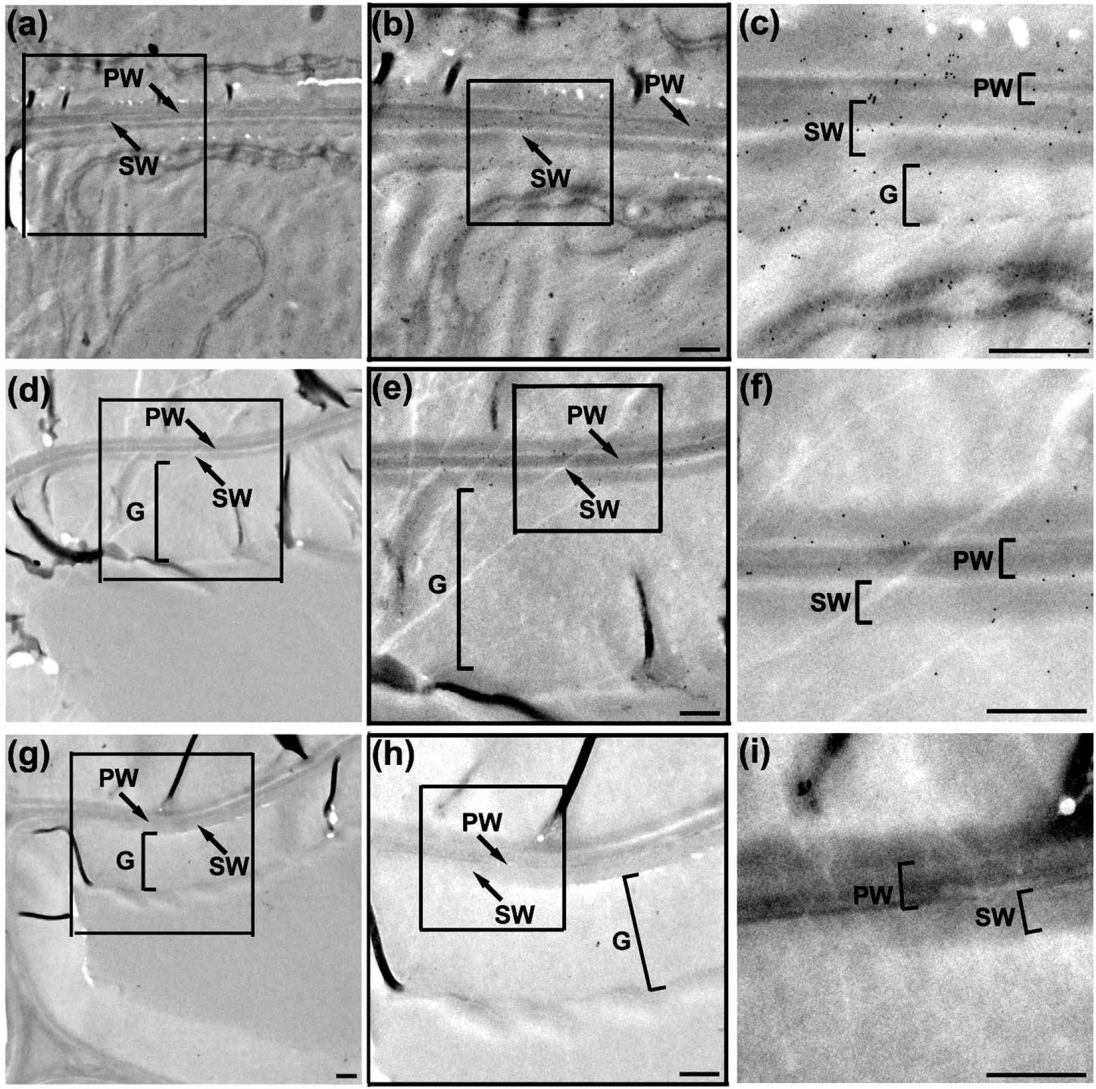
Immunocytolocalization of PtFLA6 in the G-fiber cells of TW xylem tissues. Transverse ultra thin sections of the xylem in the TW side were used for immunogold labeling TEM analysis. (**a**,**d**,**g**) Accumulation of PtFLA6 protein was examined in the differentiating G-fibers (**a**), the mature G-fibers (**d**) and the negative control (**g**), respectively. (**b**,**e**,**h**) Higher magnification images of the framed areas in (**a**,**d**,**g**), respectively. PtFLA6 labeling (black dots) was mainly detected in the differentiating G-fibers compared to that in the mature G-fibers. No labeling was seen in the control. (**c**,**f**,**i**) Higher magnification images of the framed areas in (**b**,**e**,**h**). PW, primary wall; SW, secondary wall; G, G-layer. Scale bars = 250 nm.

To confirm the location of PtFLA6 proteins in the cell wall of GF (G-fiber), immunogold labeling TEM analysis was performed. During TW formation, G-layer began to form in the DGF (Fig. 3a) which then developed into mature GF containing three distinct layers: primary wall (PW) with high electron opacity, secondary wall (SW) and thick G-layer with low electron opacity (Fig. 3d,g). The immunogold signals of PtFLA6, shown as black dots, were abundant in cell wall of DGF as well as in cytoplasm (Fig. 3b). However, the signals significantly decreased in MGF and mainly presented in PW and SW compared with the G-layer (Fig. 3e). The signals were more obvious under higher magnification of the images (Fig. 3c,f). In the negative control, no signal was observed (Fig. 3g–i). All these results indicate that PtFLA6, as a TW-associated protein, may mainly function in the G-fiber differentiation during TW formation.

### Antisense expression of *PtFLA6* inhibits TW formation

**Figure 4:**
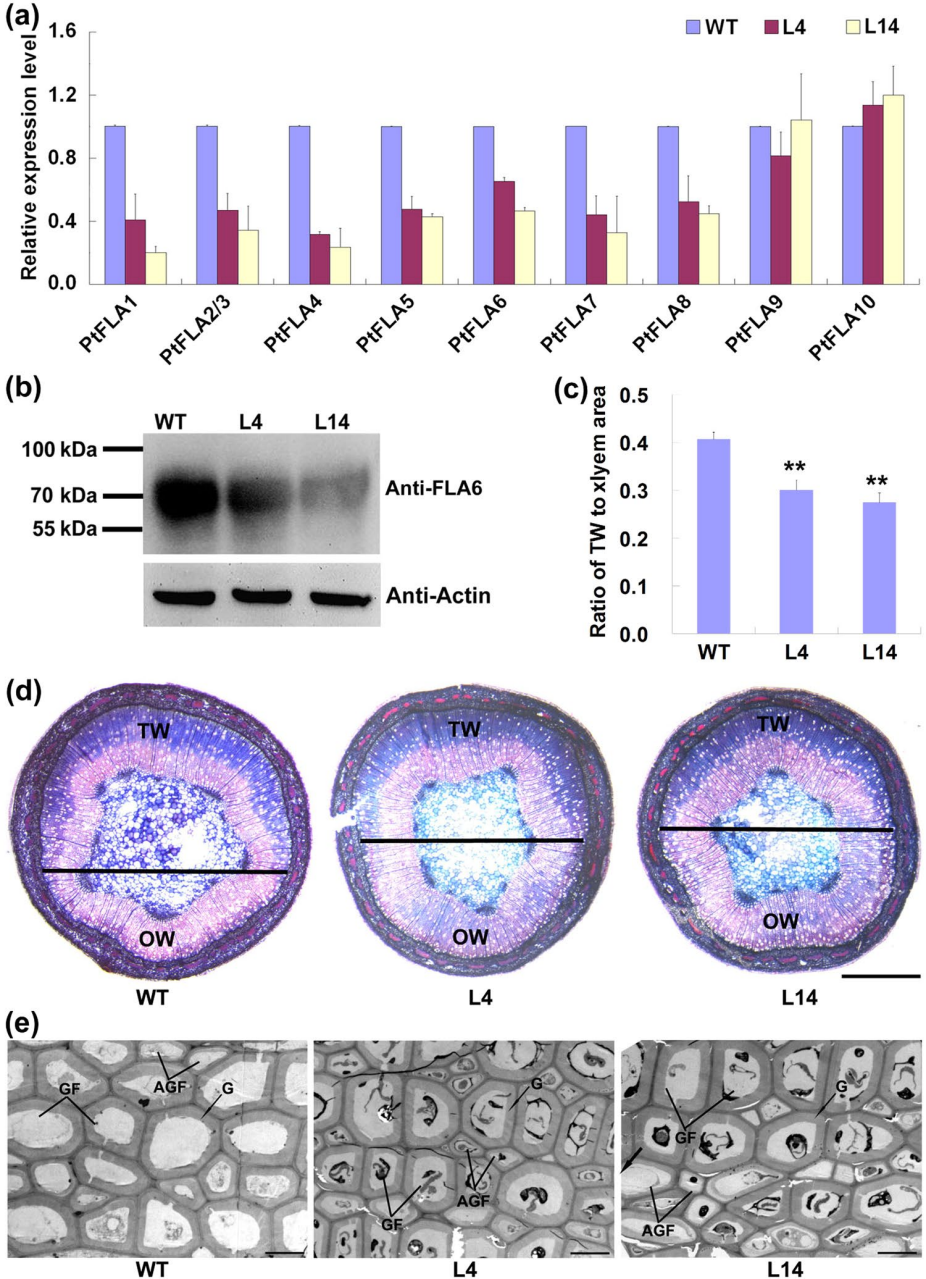
TW formation was inhibited in *PtFLA6* antisense transgenic plants. (**a**) Expression analysis of *PtFLAs* in the transgenic TW xylem. The expression of *PtFLAs* in the TW xylem of WT was set to 1. Error bars represent SDs from three biological replicates. (**b**) Western blotting analysis of PtFLA6 protein in the transgenic TW xylem. An amount of 30 μg proteins were separated by 10% SDS-PAGE and hybridized with PtFLA6 antibodies (Anti-FLA6) or plant actin antibodies (Anti-Actin), respectively. WT, wild type; L4 and L14, transgenic lines 4 and 14. (**c**) Ratio of TW area in the xylem area of transgenic plants. Error bars represent the SDs from five plants. **Indicates significant difference in comparison to WT at *P* < 0.01 (Student’s t-test). (**d**) Histochemical staining analysis of TW formation in transgenic plants. The horizontal line indicates the division between TW side and OW side. TW, tension wood; OW, opposite wood. Scale bars = 1 mm. (**e**) Transmission electron micrographs of G-fibers in transgenic TW. More AGF were present in transgenic TW. GF, G-fiber; AGF, abnormal G-fiber﻿ with stunted G-layer production; G, G-layer. Scale bars = 10 μm.

To illustrate the role of PtFLAs in TW formation, two-month-old *PtFLA6* antisense transgenic plants (lines 4 and 14) grown in greenhouse were bended for ten days to induce TW formation. Then, the xylem tissues in the TW side were collected for qRT-PCR analysis. Expressions of most *PtFLAs* (*PtFLA1-8*) were down-regulated and reduced by 40% to 80% in the TW xylem of transgenic poplar plants compared with that in the TW xylem of wild type (WT) (Fig. 4a), indicating that antisense silencing of *PtFLA6* could also reduce the transcriptions of *PtFLAs*. Consistently, PtFLA6 proteins were obviously decreased in the TW xylem tissues of transgenic plants (Fig. 4b). By histochemical staining, we observed that TW formation in the upper side of bended transgenic stems was inhibited, leading to a lowered TW-to-xylem area ratio (Fig. 4c,d). We further investigated the G-fibers in the defined position of TW tissues by TEM analysis. The results showed that most mature G-fibers of WT plants contained a thick G-layer. However, more abnormal G-fibers (AGFs) with a thin G-layer were present in the TW tissues of transgenic plants (Fig. 4e). In addition, the G-layer thickness of mature GF was also measured, and no significant difference was observed between WT and transgenic plants (Figure S2). All these results reveal that PtFLAs play important roles in TW formation and differentiation.

### GA_3_ biosynthesis and GA signaling are enhanced in TW xylem

**Figure 5:**
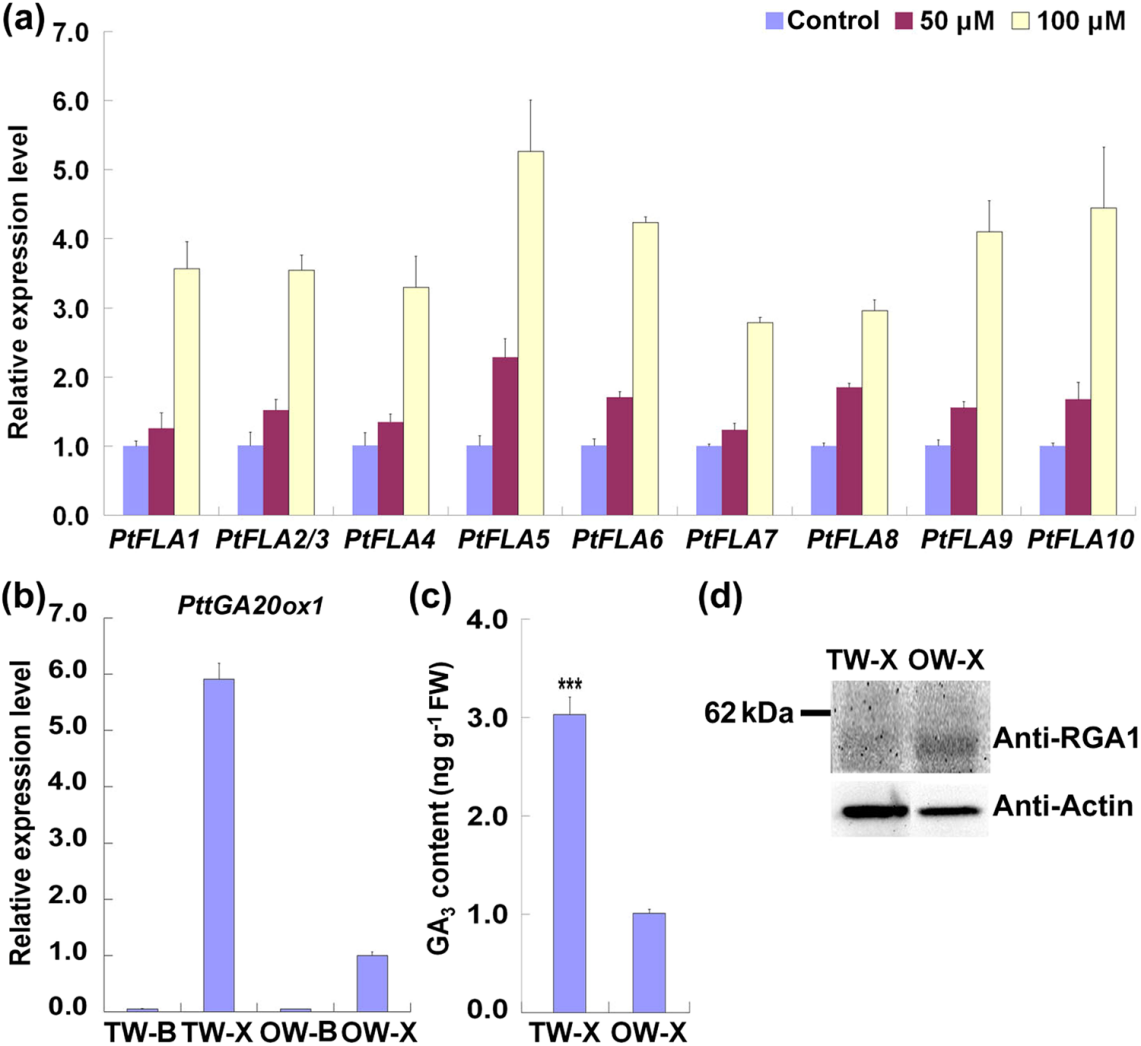
Asymmetrical distribution of GA_3_ during TW formation. (**a**) Expression analysis of *PtFLAs* in GA_3_ treated poplar stem sections. The expression level of genes in control was set to 1. (**b**) qRT-PCR analysis of *PttGA20ox1* during TW formation. Gene expression in the OW xylem was set to 1. (**c**) GA_3_ content in the xylem tissues of the TW and OW sides. ***Indicates a significant difference in comparison to OW-X at *P* < 0.001 (Student’s t-test). (**d**) Western blotting analysis of the poplar DELLA protein in the xylem of TW and OW sides. About 30 μg proteins were separated by 10% SDS-PAGE and hybridized with AtRGA1 antibodies (Anti-RGA1) or plant actin antibodies (Anti-Actin), respectively. TW-B, the bark tissues of TW side; TW-X, xylem tissues in the TW side; OW-B, the bark tissues of OW side; OW-X, xylem tissues in the OW side. Error bars represent the SDs from three biological replicates.

To illuminate how *PtFLAs* were regulated during TW formation, we further analyzed the regulatory elements in their promoters. GA responsive elements were commonly found in the promoters of most *PtFLAs* (data not shown). More importantly, expressions of all *PtFLAs* (*PtFLA1*-*10*) were significantly induced by exogenous GA_3_ treatment (Fig. 5a), indicating that GA_3_ may be an upstream regulator of *PtFLAs* during TW formation. To demonstrate this supposition, we first confirmed whether GA_3_ was a direct hormonal regulator involved in TW formation. By analyzing the gene expression pattern, we found that *PttGA20ox1*, a key endogenous GA biosynthesis gene^23^, was highly up-regulated in the TW xylem compared with that in the OW xylem (Fig. 5b). Meanwhile, GA_3_ content increased about two folds in the TW xylem compared to that in the OW xylem (Fig. 5c). This result was also confirmed by immunohistochemical test using GA_3_ monoclonal antibody, showing that GA_3_ signals in the TW tissues were obviously stronger than that in the OW tissues (Figure S3).

Increased GA_3_ content during TW formation may have triggered the GA signaling. In plants, GA signaling is mediated by DELLA proteins which function as the conserved repressors in GA-induced growth and development^24, 25^. Therefore, we investigated the protein content of the poplar DELLA proteins using AtRGA1 antibody. AtRGA1, as the key DELLA protein in *Arabidopsis*, is the homology of a putative *Populus* DELLA protein PtRGA1. Before performing the Western blotting, we examed the AtRGA1 antibody and found that it could successfully identify the purified PtRGA1 recombinant protein (Figure S4). Western blotting analysis using bended stems of wild type plants demonstrated that PtRGA1 was obviously reduced in the TW xylem tissues compared with that in the OW xylem tissues, implying an enhanced GA signaling during TW formation (Fig. 5d). These results suggest that GA and GA signaling may play crucial roles in TW formation.

### TW formation and *PtFLA* expression are inhibited by a GA biosynthesis inhibitor

**Figure 6:**
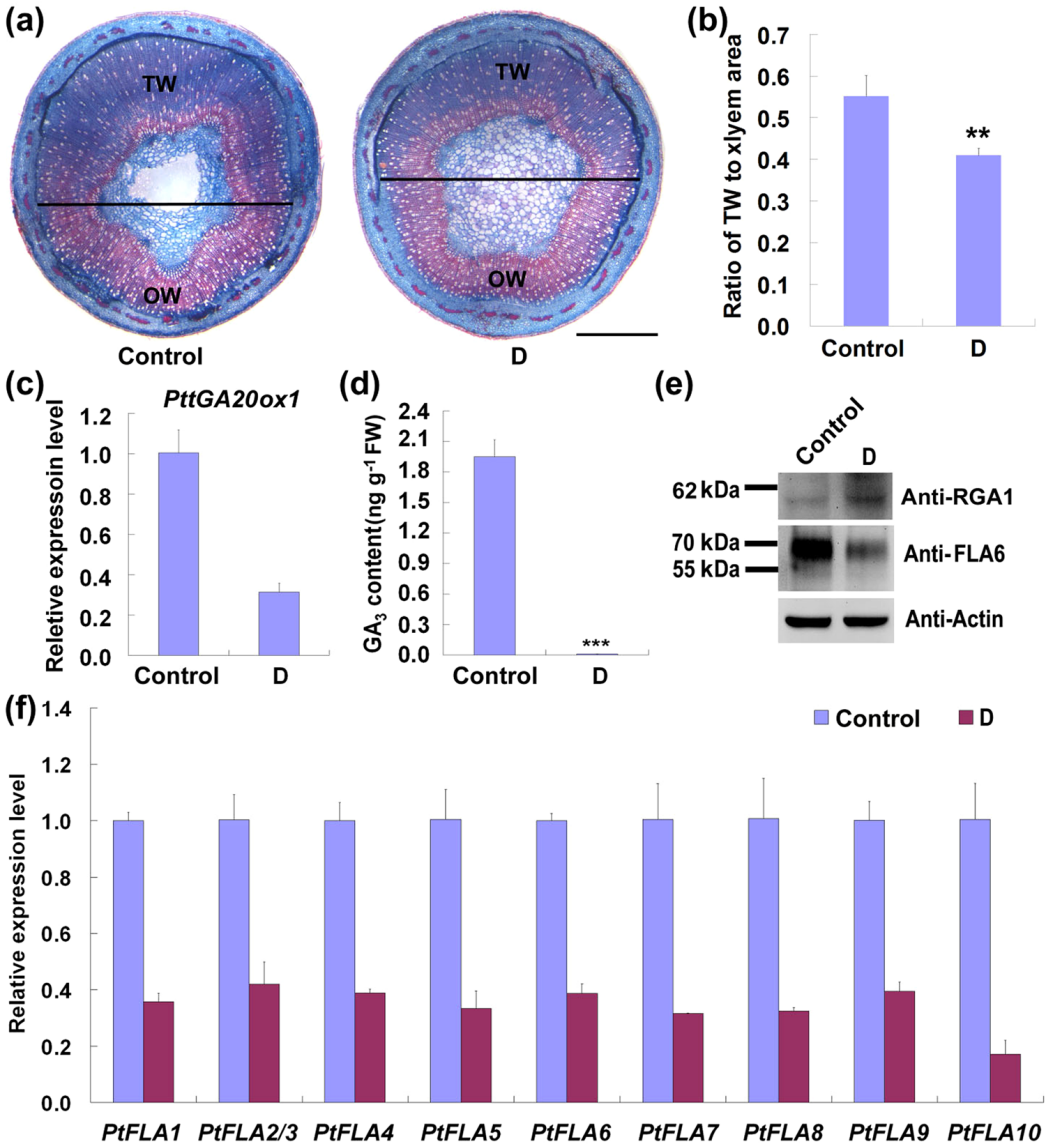
TW formation was inhibited by daminozide treatment. (**a**) Cross-sections of bended stems treated with daminozide or lanolin alone. The horizontal line indicates the division between TW side and OW side. TW, tension wood; OW, opposite wood. Scale bars = 1 mm. (**b**) Radio of TW area in the xylem of bended stems treated with daminozide. Error bars represent the SDs from five plants. (**c**) Expression analysis of *PttGA20ox1* in the TW side of bended stems treated with daminozide. (**d**) GA_3_ content in the TW side of daminozide treated stems. (**e**) Western blotting analysis of the poplar DELLA protein and PtFLA6 protein in the xylem of the TW side. An amount of 30 μg plant proteins extracted from the TW xylem tissues of bended stems treated with daminozide or lanolin were separated by 10% SDS-PAGE and hybridized with AtRGA1 antibodies (Anti-RGA1), PtFLA6 antibodies (Anti-FLA6) or plant actin antibodies (Anti-Actin). (**f**) qRT-PCR analysis of *PtFLA*s in the TW xylem tissues of bended stems treated with daminozide. Gene expression level in control was set to 1. Error bars represent the SDs from three biological replicates. Control, bended stems painted with lanolin alone; D, bended stems painted with daminozide. **Indicates a significant difference in comparison to control at *P* < 0.01 (Student’s t-test).

To investigate the roles of GA_3_ during TW formation, a GA biosynthesis inhibitor daminozide was used to treat the bended stems of wild type poplar. Histochemical staining analysis showed that TW formation was obviously repressed in the bended stems treated with daminozide, leading to a significantly reduced TW-to-xylem ratio (Fig. 6a,b). Then, the transcriptional level of *PttGA20ox1* and the content of GA_3_ were analyzed. We found that, accompanying a severe reduction in GA_3_ content, *PttGA20ox1* expression was significantly decreased in the TW side of bended stems treated with daminozide (Fig. 6c,d). In addition, Western blotting results showed that more PtRGA1 proteins were accumulated in the TW xylem of the daminozide treated stems than in that of the control (Fig. 6e). All these results demonstrate that GA plays an important role in regulating poplar TW formation.

Based on the result that *PtFLAs* were induced by exogenous GA_3_ (Fig. 5a), we speculated that expressions of *PtFLAs* could be repressed by daminozide treatment. As we expected, PtFLA6 protein content was obviously decreased in TW xylem tissues of the daminozide treated stems (Fig. 6e). Meanwhile, the transcription levels of all *PtFLA* genes were decreased in the tissues of bended stems treated with daminozide (Fig. 6f). All these results indicate that GA can regulate the expression of *PtFLAs* during poplar TW formation.

### PtRGA1 negatively regulates *PtFLAs*

**Figure 7:**
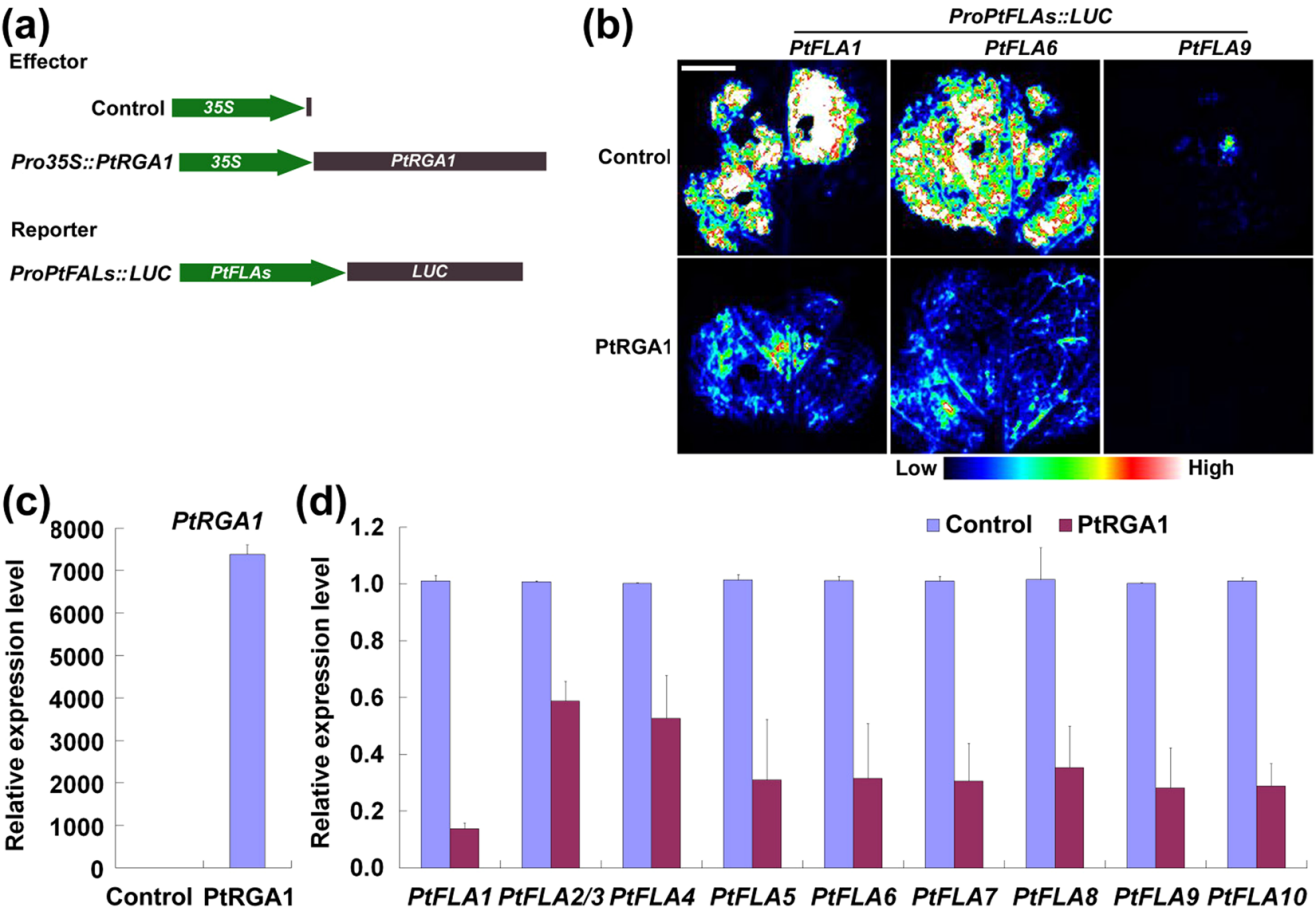
PtRGA1 negatively regulates the expression of *PtFLAs*. (**a**) Diagrams of effector and reporter constructs for transactivation studies. (**b**) Transactivation assays. Promoter activities of *PtFLA1*, *PtFLA6* and *PtFLA9* were obviously repressed in *N*. *benthamiana* leaves infiltrated with the construct combinations of *Pro35S::PtRGA1* and each *ProPtFLA::LUC*. Bars = 1 cm. (**c**,**d**) Expression analysis of *PtFLA*s in the poplar xylem protoplasts overexpressing *PtRGA1*. RNA was extracted from the poplar xylem protoplasts transfected with pGreenII62-SK-*PtRGA1*. Then qRT-PCR was performed to analyze the transcriptional levels of *PtRGA1* (**c**) and *PtFLA*s (**d**). Gene expression level in control was set to 1. Error bars represent the SDs from three biological replicates. Control, protoplasts transfected with pGreenII62-SK; PtRGA1, protoplasts transfacted with pGreenII62-SK-*PtRGA1*.

To understand how the expression of *PtFLAs* is regulated by GA signaling, transient expression assays were performed. We found that the activity of *PtFLA* promoters was repressed by PtRGA1. When the effector of *PtRGA1* was co-expressed with each reporter of *PtFLA* promoters (*PtFLA1*, *PtFLA6* and *PtFLA9*) in tobacco leaves, the fluorescence signals were weaker than that of the control (Fig. 7a,b). In addition, by the transient expression of *PtRGA1* in poplar xylem protoplasts, we found that transcriptional levels of all *PtFLAs* were obviously decreased in the transfected protoplasts overexpressing *PtRGA1* (Fig. 7c,d). These results imply that the poplar DELLA protein PtRGA1 negatively regulates the expression of *PtFLAs*.

## Discussion

When TW was produced in the upper side of bended stems, the expression of *PtFLAs* was significantly increased in the TW xylem tissues compared to that in the OW xylem and the xylem tissues of normally grown plants (Figure S1). These results are highly consistent with previous reports about the expression pattern of the TW-associated FLA genes in poplar^7, 18–20, 26^. To date, the tissue and cellular locations of poplar TW-associated FLAs have not been well reported. PtFLAs shared high identity in their amino acid sequences and similar size of protein molecular weight^21^, indicting that PtFLA6 polyclonal antibody may be able to identify all PtFLA6-like proteins, including other PtFLAs. Using PtFLA6 polyclonal antibody, we investigated the exact position of PtFLAs during TW by immunohistochemical analyses. PtFLA6 like proteins were abundantly accumulated in the DGF undergoing G-layer biosynthesis, and decreased as G-layer matured (Fig. 2c–g). This result was supported by the findings of more accurate analyses using immunogold labeling TEM of PtFLA6 (Fig. 3). The reduction of PtFLA proteins in mature GF is also consistent with a previous report showing that AGPs are strongly labeled in the secondary cell wall and less present in the G-layer of TW xylem fibers^18^. In addition, we also observed that the molecular weight of mature PtFLA6 detected in poplar stems was larger than that of its native protein. This could be caused by the glycosylation during the maturing of PtFLA protein. Indeed, five N-glycosylated sites and eleven O-glycosylated sites were predicted in PtFLA6 using the network prediction software^21^.

Although the TW-associated FLAs in trees have been identified for decades, their biological functions are still unknown. Using the bended *PtFLA6* antisense transgenic poplar grown in greenhouse, we found that PtFLAs play important roles in TW formation and differentiation. Reducing the expressions of *PtFLAs* and PtFLA6 proteins in the xylem tissues of the TW side resulted in a repressed TW formation (Fig. 4a–d) and more AGFs with thinner G-layer (Fig. 4e). These results are consistent with the TW-specific expression pattern of *PtFLA* genes and the location of PtFLA6 like proteins in DGF during TW formation. However, PtFLAs did not affect the G-layer thickness (Figure S2). Although this has fallen out of our expectation, it is easy to understand based on the protein location test showing that PtFLA6 proteins were less presented in the G-layer of MGF (Fig. 3). It is note worthy that even the expressions of most *PtFLAs* were reduced by 40% to 80% in the TW tissues of *PtFLA6* antisense transgenic lines (Fig. 4a), TW formation was not severely or completely inhibited. This could be due to the redundant functions of PtFLAs, whose CDS are highly conserved^21^, and the residual transcripts of *PtFLAs* (*PtFLA1*/*2*/*3*/*5*/*6*/*7*/*9*/*10*), which are abundant enough for TW formation in transgenic plants.

It is widely considered that the G-layer, in the inner side of TW xylem, is a central part to generate the tensile force to push the inclined stem back to the vertical position. Using three-point tests, we also found that the flexural strength and stiffness of the bended stems with TW formation were all decreased in transgenic plants compared to that in WT plants (Figure S5). FLAs contain one or more cell adhesion domains (fasciclin domain), which work as adhesion molecules between the macromolecules of secondary cell wall by attaching to the plasma membrane^13^. We speculate that PtFLAs located in the DGF may be responsible for the tensile force in the outer side of TW xylem.

As an important class of plant hormones, GA participates in the differentiation and elongation of xylem fibers and lateral root formation in trees^27–31^. Previous studies have found that GA could play a positive role during TW formation, based on the observations that TW formation was induced or inhibited by the treatments using exogenous GA or GA biosynthesis inhibitors^11, 32–34^. We found that GA_3_ and *PttGA20ox1* transcripts were asymmetrically distributed in the upper side of bended stems undergoing TW formation (Fig. 6b,c; Figure S4). These results are similar to the previous report that strong expression of *PttACO1* (an ACC oxidase gene) and ethylene biosynthesis activity in TW xylem were detected^9^. Furthermore, daminozide treatment could inhibit GA_3_ biosynthesis by repressing the expression of *PttGA20ox1* in the TW tissues, resulting in a severe inhibited TW formation (Fig. 6a–d). Correspondingly, TW formation was also obviously suppressed in poplar plants treated with an ethylene perception inhibitor (1-methylcyclopropene, 1-MCP)^10^. Therefore, GA may be another important plant hormone involved in regulating poplar TW formation. In addition, the transcription levels of *PtFLAs* were induced by exogenous GA_3_ treatment (Fig. 5a), which is coincident with a recent report showing that GA could promote the expressions of poplar *FLAs* ^7^. Our data revealed that the high level of GA_3_ in the TW tissues of the bended stems or the decreased level of GA_3_ in the inhibited TW by daminozide treatment was accompanied with up- or down-regulated *PtFLAs*, suggesting that GA_3_ is an upstream regulator of *PtFLAs*.

The next question is how GA_3_ regulates *PtFLAs*? As we know that GA signaling is mediated by the conserved repressors: DELLA proteins^24, 25^. GAs bind to the GA receptor GIBBERELLIN INSENSITIVE DWARF1 (GID1) to form a GA-GID1 complex, which immediately triggers the rapid degradation of DELLA proteins, then GA-induced genes are activated to complete the GA-regulated plant development and growth^35^. The relationship between the repressed TW formation by daminozide, which severely reduced GA_3_ content, and the inhibited degradation of PtRGA1 suggests that GA_3_-regulated TW formation is meditated by the DELLA protein PtRGA1 (Fig. 6a–e). DELLAs act as repressors in GA-mediated responses by interacting with other transcription factors and blocking their DNA binding or transactivation activities^36^. However, DELLA-DNA interaction may be indirect or less efficient by analyzing the RGA binding region of the promoter of its target genes using ChIP-qPCR test^37^. Therefore, we performed tobacco transient expression assays and overexpressed *PtRGA1* in xylem protoplasts to demonstrate the negative regulation of *PtFLAs* by PtRGA1.

**Figure 8:**
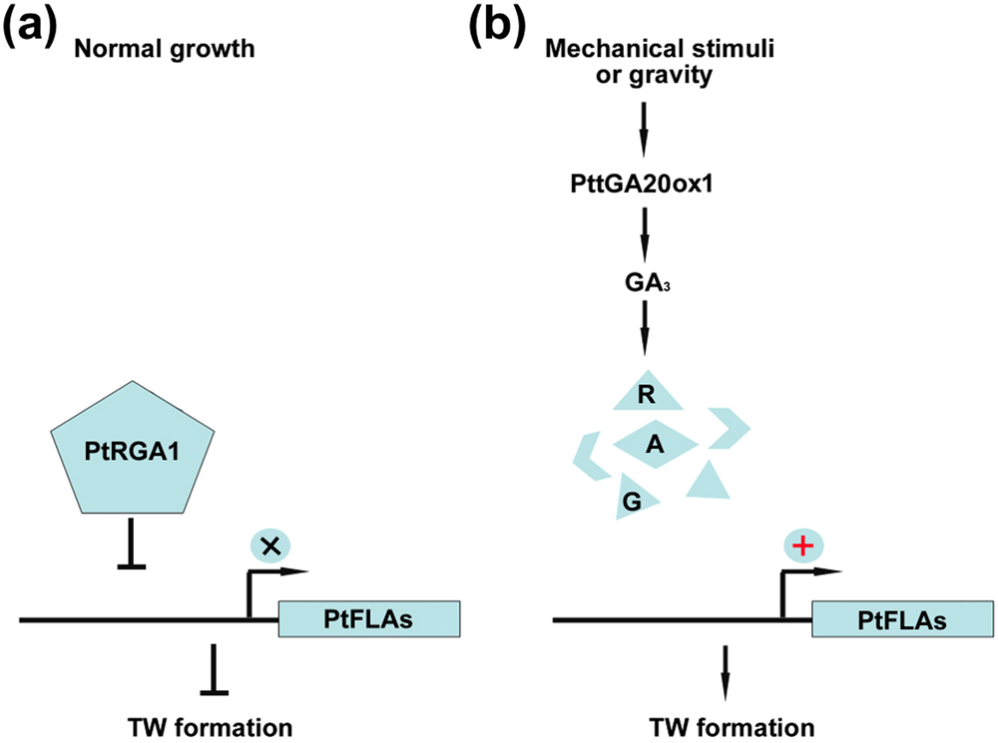
A proposed model of GA regulated TW formation. (**a**) The poplar DELLA protein PtRGA1 inhibits the expression of *PtFLAs* under normal growth, and then represses the TW formation. (**b**) When submitted to mechanical or gravity stress, more GA_3_ will be produced on the upper side of the bended or tilted poplar stems, and then triggers the degradation of PtRGA1 proteins, which promotes *PtFLAs* transcription to facilitate TW formation.

Taken together, this study illustrates the biological function of PtFLAs in TW formation, as well as the regulatory mechanism of GA to *PtFLAs*. Although, more evidence is needed to completely understand the mechanism of GA and its signaling in TW formation, our data presented here imply a possible model of TW formation: under normal growth condition, the poplar DELLA protein PtRGA1 suppresses the transcriptions of *PtFLAs*, and then inhibits the TW formation (Fig. 8a); when poplar plants are subjected to mechanical or gravity stresses, more GA_3_ is produced in the xylem of upper side of bended or tilted stems to trigger the degradation of PtRGA1, and as a result, expressions of *PtFLAs* are activated to facilitate TW formation (Fig. 8b).

## Methods

### Plant materials and growth conditions

*Populus trichocarpa* genotype Nisqually-1 was used for the cloning of genes and promoters. Wild type Shanxin yang (*P*. *davidiana × P*. *bolleana*), a commercial hybrid clone, was used in every experiment as control in this study. The *PtFLA6* antisense transgenic plants were generated by transforming the antisense *PtFLA6* vector into Shanxin yang as described in our previous report^21^. All poplar plants were grown in individual pot in a greenhouse at 25 °C (day)/18 °C (night) in a 12 h light/12 h dark photoperiod. Tobacco plants (*Nicotiana benthamiana*) were grown in the greenhouse at 21 °C (day)/19 °C (night) under long-day condition (16 h of light/8 h of dark).

### TW induction and histochemical staining

Before the induction of TW, WT and transgenic poplar plants grown in greenhouse were fixed to the sticks to make sure all the plants grow straight. The two-month-old poplar plants were bended at the twelfth internode (count from the top) to a 45° angle and fixed with cotton ropes to induce TW formation. After treated for 2 weeks, TW was well produced in the upper side of the bended stems and detected by histochemical staining as described below.

About 0.5 cm thick segments of bended internodes were fixed overnight at 4 °C in FAA solution. The segments were subsequently, passed over a gradient ethanol series, and then embedded in paraffin. Ten micrometer thick sections were cut out with a rotary microtome, and stained with 1% aqueous safranin-O (MP, USA) and subsequently with 1% aqueous astra-blue (Santa Cruz, USA) as described previously^38^. TW was stained into blue color in the xylem tissues of the upper side of the bended stems. OW remained in red color in the lower side of the bended stems. Images were taken under the SMZ800 microscope (Nikon, Japan). TW area and the whole xylem area of the bended stems were measured using the microscope software NIS-Element D 3.1 (Nikon, Japan). Then, the TW to xylem area ratio was calculated to illustrate TW formation.

### Antibodies

PtFLA6 polyclonal antibody was produced by ABclonal (http://www.immunogen.com.cn) as described previously^21^. *Arabidopsis* RGA1 polyclonal antibody was purchased from Agrisera (http://www.agrisera.com/). Plant actin monoclonal antibody and the secondary antibodies used in this paper were bought from ABclonal.

### Immunohistochemical localization

Segments (0.5 cm) of the bended stems for two weeks were fixed overnight in 4% paraformaldehyde in 0.1 M phosphate-buffered saline (PBS, pH 7.5). Sections (10 µm) were cut out and blocked with 5% bovine serum albumin (BSA) for 1 h at room temperature. Then the sections were incubated with the PtFLA6 antibody (diluted at 1:50 with 0.1 M PBS containing 0.1% BSA) at room temperature for 1 h. After rinsed three times in PBS (5 min each), the samples were immersed in the alkaline phosphatase-conjugate secondary antibody (diluted at 1:100) for 1 h and then stained with 150–200 µl of Western Blue (Promega, USA). When blue color appeared, images were captured under bright field using the ECLIPSE 80i microscope (Nikon, Japan) or SMZ800 microscope (Nikon, Japan). Pre-immune serum was used as the negative control.

### Quantitative real-time RT-PCR

For expression analysis of *PttGA20ox1* during TW formation, the bended stems were cut into two parts from the division of TW side and OW side as shown in Fig. 1b. Then the bark tissues stripped from the TW part and OW part (TW-B or OW-B) as well as the remnant xylem tissues with a part of pith (TW-X and OW-X) were collected to extract total RNA with the RNAiso Reagent (Takara, Japan). To detect the expression level of *PtFLAs* in transgenic plants during TW formation, the xylem tissues separated from the TW side were for RNA extraction. A total amount of 2 μg RNA was subjected to reverse transcription reaction using the HiScript® II Q RT SuperMix for qPCR (+gDNA wiper) (Vazyme, China). qRT-PCR was performed using a AceQ qPCR SYBR Green Master Mix (Vazyme, China) and a CFX Connect Real-Time System (Bio-Rad, USA). The elongation factor gene *PtEF1β* was employed as an internal control in this study. And the relative expression of each target gene was normalized using *PtEF1β*. Gene specific primers used in this study were listed in Table S1. *PttGA20ox1* primers were used as described previously^23^.

### PtFLA6 promoter activity analysis

To construct the promoter-GUS vector, the *PtFLA6* promoter (~1.5 kb) was cloned from Nisqually-1 and inserted into in pBI121 by replacing the 35S promoter. The resultant construct was introduced into Shanxin yang by *Agrobacterium*-mediated transformation as described previously^39^. The segments of bended stems of *PtFLA6* promoter-GUS transgenic plants were used for GUS staining as described previously^39^. Then the segments were fixed in FAA solution and embedded in paraffin. Sections (10 µm) were cut out and observed under bright field with the SMZ800 microscope (Nikon, Japan).

### Western blotting

Total proteins were extracted using RIPA buffer, separated by 10% sodium dodecyl sulfate-polyacrylamide gel electrophoresis (SDS-PAGE), and then electrotransfered onto polyvinylidene difluoride membranes. After blocked in TBST buffer supplemented with 5% non-fat dried milk for 1 hour at room temperature, the membranes were incubated with anti-PtFLA6 antibodies or anti-RGA1 (diluted at 1:1000) for 1 hour at room temperature. Afterwards, the membranes were rinsed three times with TBST buffer and subsequently incubated with the secondary antibodies (at a dilution of 1:5000) for 1 hour at room temperature. Then the membranes were put in the chemiluminescence detection solution LumiGLO (KPL, USA) and detected using the chemiluminescence image system Tanon 5500 (Tanon, China).

For western blotting analyses of PtFLA6 and poplar RGA1 during TW formation, xylem (X) and bark (B) tissues in the TW and OW sides of WT were separated for protein extraction. To detect PtFLA6 accumulation in transgenic TW, TW xylem tissues were used for protein extraction.

### TEM and immunogold labeling TEM

For TEM (transmission electron microscopic) observation, TW tissues from the bended stems were fixed in glutaraldehyde solution, and embedded in EPON 812 resin (Shell, Holland). Sections (80 to 70 nm) were cut out, post-stained with uranyl acetate and lead citrate, and then observed with an H-7650 electron microscope (HITACHI, Japan).

For immunogold labeling TEM analyses, sections on grids were incubated with anti-PtFLA6 antibodies (diluted at 1:50). After hybridized with goat anti-rabbit gold conjugate (10 nm; Sigma, USA), the grids were washed and stained with 2% aqueous uranyl acetate. Control sections were obtained by omitting the primary antibody.

### GA_3_ content assays

GA_3_ extraction was performed as described previously with minor modification^31^. Initially, freeze-dried materials were extracted with 80% (v/v) methanol containing the internal standard of [^2^H_2_]-GA_3_. The aqueous phase was extracted and then purified on C_18_ Sep-Pak and MCX SPE columns (Qasis; Waters, USA). The eluant was dried, re-dissolved in HPLC initial solution, and then detected with a liquid chromatography-mass spectrometry system (Thermo, USA). Tandem mass spectrometry data were analyzed using the Xcalibur 2.1 software (Thermo, USA).

### Exogenous GA_3_ and daminozide treatments

Stem sections about 1 mm thick were sliced from the upper part stems of two-month-old wild type poplar grown in greenhouse. Then, the sections were soaked in 50 and 100 μM GA_3_ aqueous solution, respectively. After 4 hours, the stem sections were used for qRT-PCR analysis of *PtFLA* expressions. Sections treated with water were used as control.

For GA biosynthesis inhibitor treatments, lanolin containing 5 mg ml^−1^ daminozide (Sigma, USA) was painted on the upper face of the bended stems of wild type plants. After two weeks, the treated stems were used for histochemical staining. Meanwhile, the TW side tissues were cut out and used for qRT-PCR, GA_3_ content determination, and Western blotting. The bended stems pained with lanolin alone were used as control.

### Tobacco transient expression assay

The promoters (~1.5 kb) of *PtFLA1*, *PtFLA6* and *PtFLA9* were amplified from the genomic DNA of Nisqually-1 and inserted into pGreenII0800-LUC^40^, respectively. The poplar DELLA protein gene *PtRGA1*, the homology of *AtRGA1*, was cloned from cDNA of Nisqually-1 to construct the pCAMBIA-2301 overexpression vector, driven by two copies of cauliflower mosaic virus 35S promoter^41^. The constructs were introduced into *Agrobacterium tumefaciens* GV3101 (pMP90) by the freeze-thaw method.

Tobacco transient expression assays were performed as previously described^42^. *Agrobacterium* was re-suspended in infiltration buffer (10 mM methylester sulfonate, 10 mM MgCl_2_, and 150 μM acetosyringone, pH 5.7) at OD600 = 0.8 and then infiltrated into the tobacco leaves. After 3 days, Luciferin (1 mM) was painted on the transfected leaves before LUC activity was monitored. LUC signal was photographed with a chemiluminescence image system Tanon 5500 (Tanon, China). Three independent experiments (biological triplicates) were performed.

### Transient expression of PtRGA1 in poplar xylem protoplasts

PtRGA1 CDS was inserted into pGreenII62-SK driven by the CaMV 35S promoter^40^. Then, stems of two-month-old poplar plants normally grown in greenhouse were used to isolate protoplasts. Xylem protoplasts isolation and cells transfection were performed as described previously^43^. Transfected protoplasts were used to for RNA extraction and qRT-PCR analyses of *PtFLAs* as described above.

## Electronic supplementary material

Supplementary information
