# Supplementary material for: Fasciclin-like arabinogalactan proteins, PtFLAs, play important roles in GA-mediated tension wood formation in Populus
Source: Sci Rep. 2017 Jul 21;7:6182. doi: 10.1038/s41598-017-06473-9 (PMC5522414; doi:10.1038/s41598-017-06473-9)
Supplement: Supplementary file 1 — Supplementary information [file 41598_2017_6473_MOESM1_ESM.doc]

Fasciclin-like arabinogalactan proteins, PtFLAs, play important roles in GA-mediated tension wood formation in *Populus*

Haihai WANG^1,4^, Yanli JIN^1^, Cuiting WANG^1^, Bei LI^1,2^, Chunmei JIANG^1^, Zhencang SUN^2^, Zhiping ZHANG^1^, Fanjing KONG^3^ and Hongxia ZHANG^1,2*^

**Supplementary information:**

**Table S1.** Primer sequences used in this study.

**Figure S1.** Expression analysis of *PtFLAs* during TW formation.

**Figure S2.** G-layer thickness of GF in TW of transgenic plants.

**Figure S3.** Asymmetric GA_3_ localizations during TW formation as detected by immunostaining with a GA_3_-specific antibody.

**Figure S4.** Purification of PtRGA1 recombinant protein and Western blotting analysis using anti-RGA1.

**Figure S5.** Biomechanical properties of the bended stems of transgenic plants.

**Table S1.** Primer sequences used in this study.

| **Gene** | **Identifier** | **Forward Primer (5' to 3')** | **Reverse Primer (5' to 3')** |
| --- | --- | --- | --- |
| **Primer for RT-PCR** | | | |
| PtEF1β | Potri.009G018600 | GACAAGAAGGCAGCGGAGGAGAG | CAATGAGGGAATCCACTGACACAAG |
| PtFLA1 | Potri.019G120900 | ATGAAGCCACAGTACTTACTCTCTT | TGGTTAAACCAGTGCTTGAATC |
| PtFLA2/3 | Potri.013G151500 | ACCAATACAAGTTTATCTGGCACG | CAAAACCAGACCAAACCCCAC |
| PtFLA4 | Potri.013G014200 | TCGAGCTTAATTTGCATTGGTATTT | GGGTGGATCTCAAAAGGCGA |
| PtFLA5 | Potri.019G123200 | CAACTTGCATTGGCATTTCTGA | TGCTTGAATCATTTAGTGCGGAG |
| PtFLA6 | Potri.013G151400 | GAATTGTTTCGAGCATTTTATCCC | CTGGAGGTTGTGCTACGACTACTG |
| PtFLA7 | Potri.012G015000 | GATGAAGCACCATTTTTCAGTCTTC | CAGGACCTTTTGAAACTTGTACAGG |
| PtFLA8 | Potri.009G012200 | TCTGCATAACAACGCCTTGC | ATGGGAATGACAGGAAAACGTC |
| PtFLA9 | Potri.004G210600 | GTAATGCCACCACCTACGACCC | CGTTTAGAAAGCCCGCTTTGAG |
| PtFLA10 | Potri.009G012100 | AGGTCGACAAGGTGCTAGTTCCT | CATTTGAGTCATTGGCCCCACT |
| PttGA20ox1 | AJ001326 | GCACAAGTTCTTCGACACCAG | GCAGCAACAGGGTTACCAGAG |
| **Primer for gene cloning** | | | |
| PtRGA1 | Potri.010G110700 | ATGGATGAACTTTTAGCTGTTTTG | TTAAGCAGCACCGCCAACTGG |
| **Primer for promoter cloning** | | | |
| PtFLA1 | Potri.019G120900 | CCGGAATTCCAACCATTCGGGATTAGCCT | CAGGAGCTCGCTAGAAACAATGCAAAAATGCT |
| PtFLA6 | Potri.013G151400 | AGAGAGAAAGGAAAAGGAGGTTG | GGATAAAATGCTCGAAACAATTC |
| PtFLA9 | Potri.004G210600 | CCGGAATTCTACCGGCTAGCATATAATTGCTTC | CAGGAGCTCTGAAGATTGTTGCCTCATCCTAC |


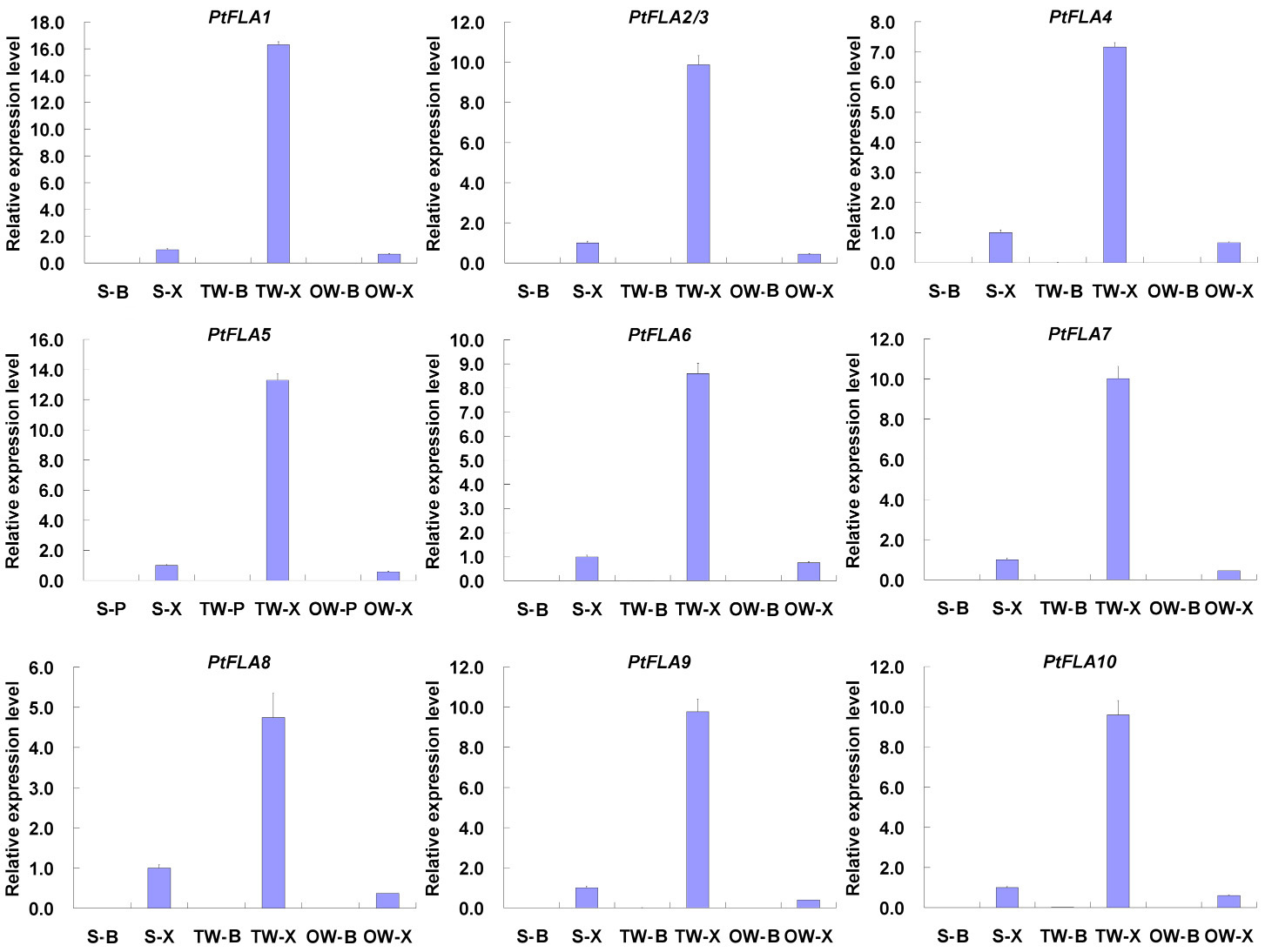


**Figure S1.** Expression analysis of *PtFLAs* during TW formation. Wide type poplar plants grown in greenhouse were used for bending treatment. The bended stems were cut to two parts from the division of TW and OW sides as shown in Figure 1b. Then barks containing the phloem tissues was stripped from the TW and OW parts respectively to represent phloem tissues (B). The remnant xylem tissues (X) with a part of pith were collected at the meantime. The six kinds of tissues: bark tissues from the vertically grown poplar plants (S-B), the TW (TW-B) and OW (OW-B) sides, xylem tissues from the vertically grown poplar plants (S-X), the TW (TW-X) and OW (OW-X) sides, were used for total RNA extraction. The expression value of xylem tissues in uprightly grown poplar plants (S-X) was set to 1. Error bars represent the SDs from three biological replicates. S-B, bark tissues in the vertically grown poplar plants; S-X, xylem tissues in the vertically grown poplar plants; TW-B, bark tissues in the TW side; TW-X, xylem tissues in the TW side; OW-B, bark tissues in the OW side; OW-X, xylem tissues in the OW side.


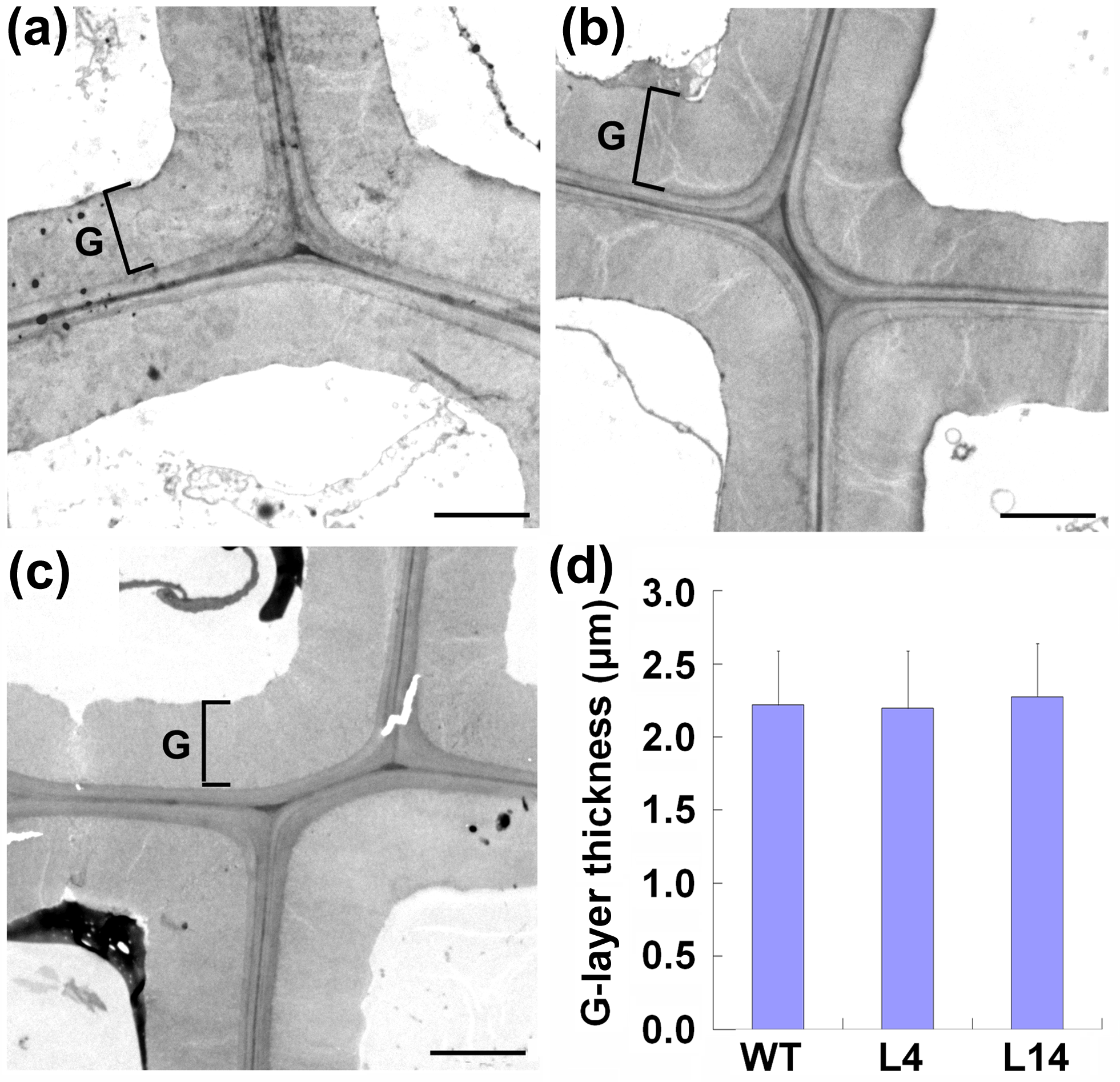


**Figure S2.** G-layer thickness of GF in the TW of transgenic plants. (a-c) G-layer fiber cells in the TW of Shanxin yang (WT), transgenic lines L4 and L14. The black lines indicate the positions where cell wall thickness was measured. G, G-layer. Scale bar = 2.5 µm. (d) Thicknesses of G-layer in the G-fibers of WT, L4 and L14. Values shown are means and SDs of 50 fiber cells from three plants of WT and each transgenic lines.


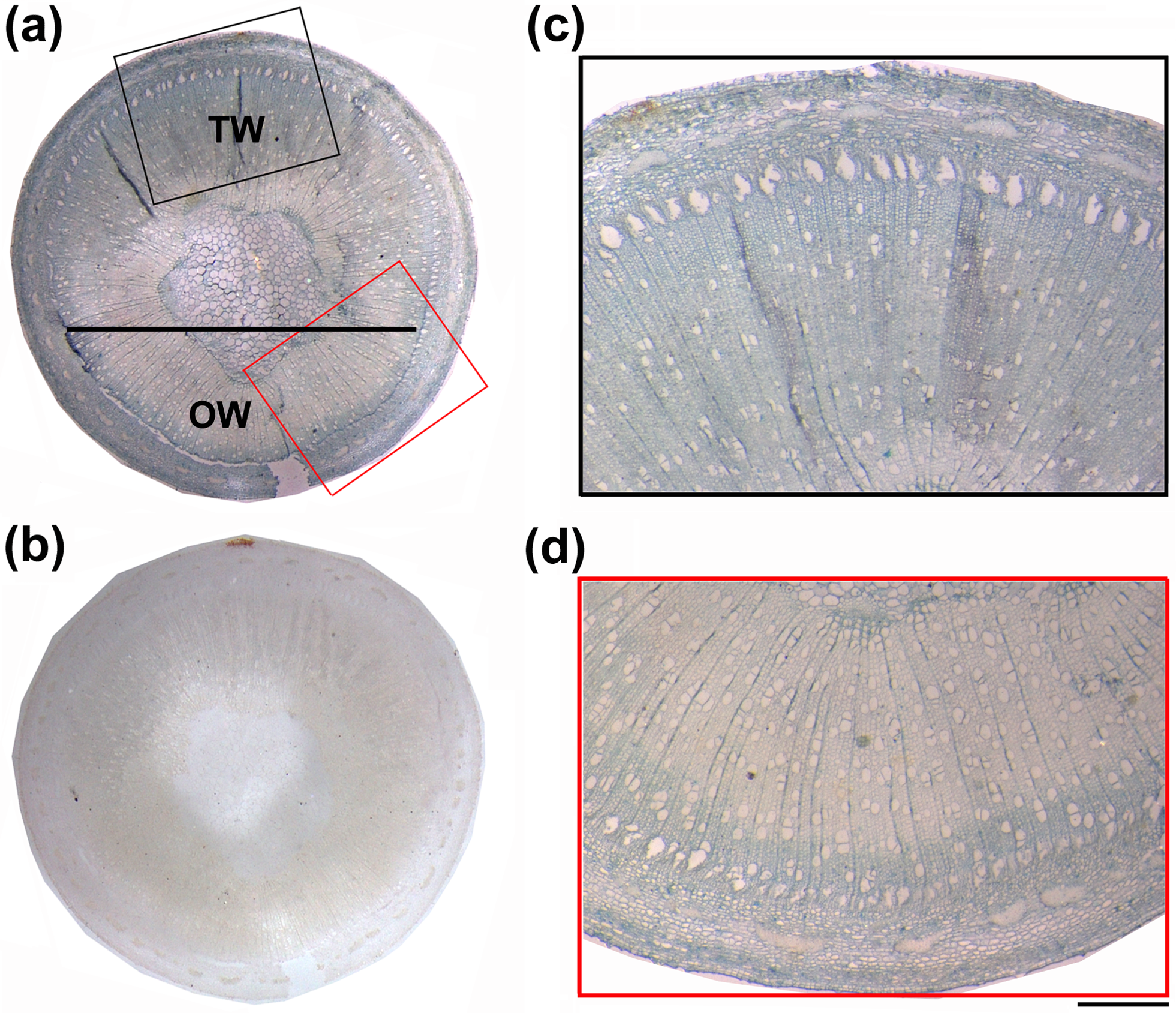


**Figure S3.** Asymmetric GA_3_ localizations during TW formation as detected by immunostaining with a GA_3_-specific antibody. (a, b) Cross-sections of the bended stems were hybridized with anti-GA3 (Agrisera, Sweden) (a) or BSA (b) as control. Blue signals of GA3 were mainly observed in the TW side compared with that in the OW side. The horizontal lines indicate the division of TW and OW. (c) A higher magnification of the black-line framed area in (a). GA3 signals (blue staining) were strongly detected in the phloem and xylem tissues of the TW side. (d) A higher magnification of the red-line framed area in (a). GA3 signals were weakly observed in the phloem and xylem tissues of the OW side. TW, tension wood; OW, opposite wood. Scale bar = 0.4 mm.


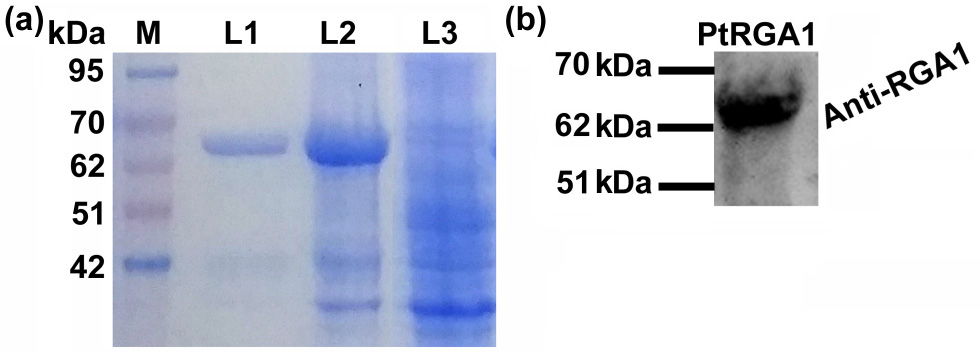


**Figure S4.** Purification of PtRGA1 recombinant protein and Western blotting analysis using anti-RGA1. (a) SDS-PAGE gel of the recombinant PtRGA1 from *Populus*. The expression vector pET-30a (+)-PtRGA1 with the gene encoding a DELLE protein driven by the IPTG-inducible promoter was introduced and expressed in *E. coli*. M, protein maker; L1, recombinant PtRGA1 purified from the IPTG-induced *E. coli* cultures; L2, protein from IPTG-induced cells; L3, extracts of non-induced cells. (b) Western blotting using anti-RGA1 whose reactivity was confirmed in *Arabidopsis*. The antibody also identified the PtRGA1 recombinant protein.

**
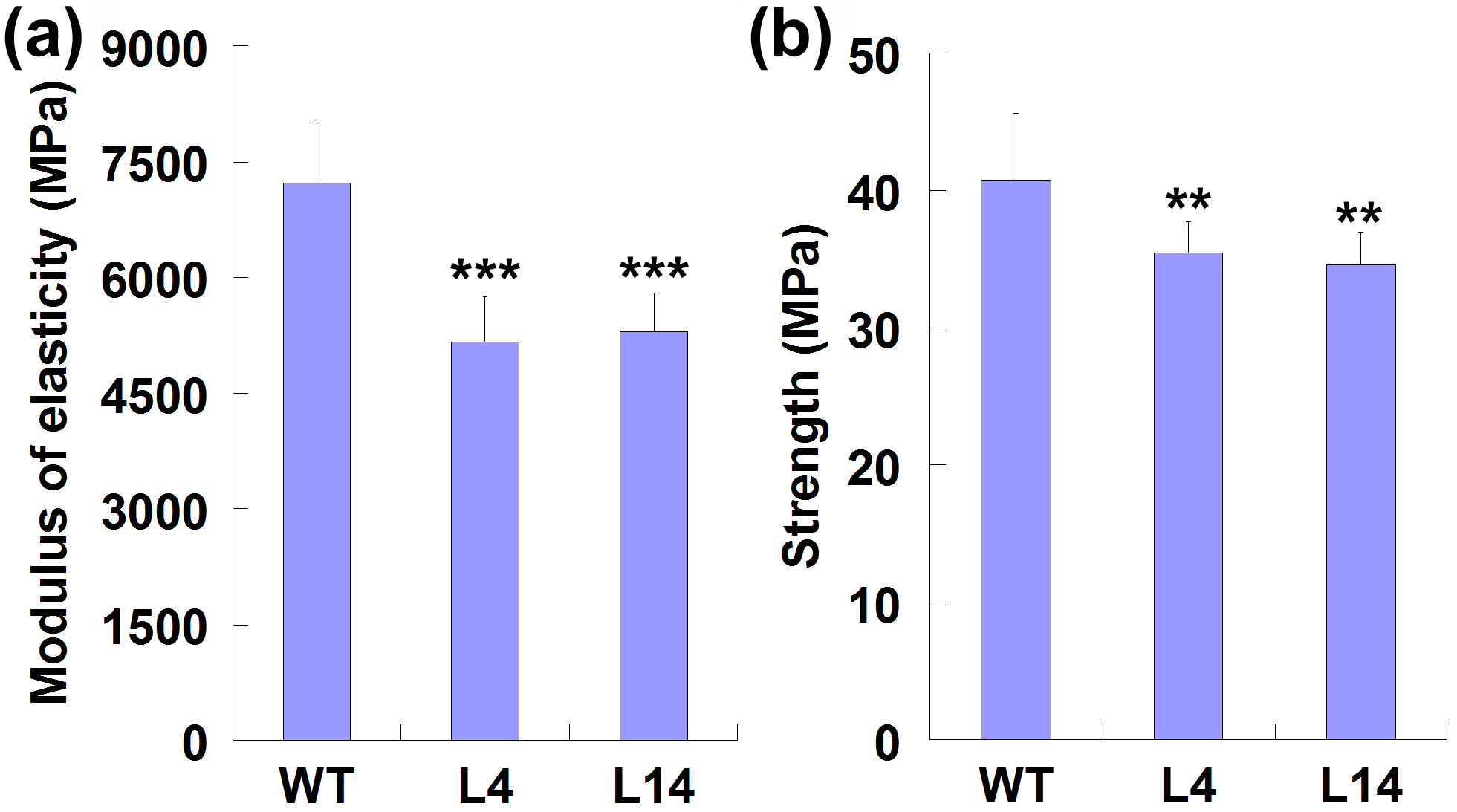
**

**Figure S5.** Biomechanical properties of the bended stems of transgenic plants. (a) Flexural stiffness test. (b) Flexural strength test. The bended stems of WT and transgenic plants were used for the test. Values are means ± SD of 10 independent plants of WT and each transgenic line. ** and *** indicate significant differences in comparison to WT at P< 0.01and P< 0.001 (Student’s t-test), respectively.
